# SURGICAL TREATMENT OF FISH IMPACTED IN THE UPPER ESOPHAGUS

**DOI:** 10.1590/0102-6720201600010018

**Published:** 2016

**Authors:** Gustavo Rêgo COÊLHO, Silvio Melo TORRES, Diego Costa de ALMEIDA, Laiza Marques MOREIRA, Thyago André Oliveira MENDES, Tiago Araújo MONTEIRO

**Affiliations:** Hospital Geral de Fortaleza, Fortaleza, Ceará, Brazil

## INTRODUCTION

The foreign body ingestion is common in emergency services. In most cases, it passes through the gastrointestinal tract spontaneously and does not cause any considerable damage. When the impaction occurs in the gastrointestinal tract, the most common level is the upper third of the esophagus. Approximately 10-20% of the cases requires endoscopic intervention and less than 1% needs some surgical procedure^4^^,^ 5. Usually, children from six months to six years old are more likely to that^3^. In adults, it is more common in individuals with psychiatric disorders, drug users, alcoholics or individuals that benefit from incident, as prisoners.

## CASE REPORT

**FIGURE1 -:**
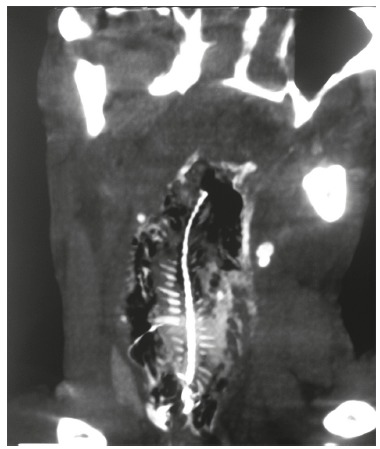
Coronal computed tomography of the neck clearly showing the fish impacted in cervical esophagus

**FIGURE 2:**
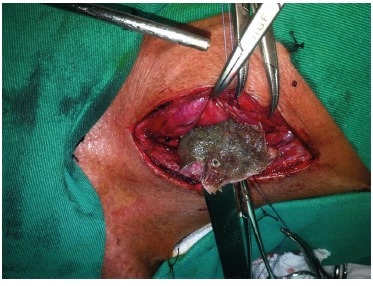
- Esophagotomy and removal of the intact fish

Male, 52, alcoholic and user of crack, previously healthy. Accidentally swallowed a Soy fish of approximately 15 cm. It quickly progressed with hematemesis and respiratory failure before medical care. At the emergency room, it was observed respiratory arrest, being promptly intubated and laryngoscopy displayed the foreign body to the cervical esophagus. He was subjected to mechanical ventilation and remained hemodynamically stable. Endoscopy was performed soon after stabilization, but without success due to an intense inflammatory process and total occlusion of the esophageal lumen by the foreign body. Cervical and thoracic computed tomography showed the whole fish on cervical esophagus (Figure 1). The patient was submitted to surgical treatment with cervicotomy and esophagotomy, removal of the fish intact and primary synthesis of esophagus with Penrose drainage (Figure 2). He evolved without complications in surgical aspect, but with myoclonus and minimum response to the existing neurological deficit, resulting from a long period of pre-hospital cerebral hypoxia.

## DISCUSSION

The greater part of foreign bodies (80%) pass through the gastrointestinal tract without difficulties, but 20% can obstruct the lumen, requiring endoscopic or surgical removal (1% of cases). As the esophagus is a narrow portion of the gastrointestinal tract, 28-68% objects are found in this region^5^. The symptoms depend on the location. Dysphagia, odynophagia and salivation suggest esophageal foreign body^4^. It can also present chest pain, cough, dyspnea, wheezing or stridor. In more severe cases, particularly in large or sharp foreign bodies, there may be intense pain, vomiting, refusal to eat, saliva ink with blood or shock^1^.

A medical review of database present several accidents involving foreign bodies ingestion, including food-bolus impactions, coins, fish bones, dental prostheses, chicken bones, iron slices, lighters, little metallic foreign bodies, toothbrushes, needles, and spoons^5^, but no reports involving the ingestion of whole fish. Impaction events with fish bones includes 12.6% of the accidents, the third highest in incidence^5^. As the majority of the bodies are radiopaque, the diagnosis can easily be done with plain radiography in posteroanterior and lateral projections. Endoscopy and contrasted study are needed in the case of radiotransparent objects. In all radiological exams it must be looked for signs of subcutaneous emphysema, which indicates drilling^3^. The treatment of choice is the endoscopic removal of the foreign body, which is successful with little or no complications for the patient^2^. The surgical treatment should be performed when endoscopic management is not possible to solve the problem, or if there is impairment of progression in the gastrointestinal tract or complications such as perforation, obstruction and bleeding^2^^,^ 3. 

## References

[1] Arana A, Hauser B, Hachimi-Idrissi S, Vandenplas Y (2001). Management of ingested foreign bodies in childhood and review of the literature. Eur J Pediatr.

[2] Brady PG (1991). Esophageal foreign bodies. Gastroenterol Clin North Am.

[3] Eisen GM, Baron TH, Dominitz JA (2002). Guideline for the management of ingested foreign bodies. Gastrointest Endosc.

[4] Hachimi-Idrissi S, Corne L, Vandenplas Y (1998). Management of ingested foreign bodies in childhood: our experience and review of the literature. Eur J Emerg Med.

[5] Zhao-Shen Li, Zhen-Xing Sun, Duo-Wu Zou, Guo-Ming Xu, Ren-Pei Wu, Zhuan Liao (2006). Endoscopic management of foreign bodies in the upper-GI tract: xperience with 1088 cases in China.

